# CPA4 as a biomarker promotes the proliferation, migration and metastasis of clear cell renal cell carcinoma cells

**DOI:** 10.1111/jcmm.18165

**Published:** 2024-03-17

**Authors:** Kongjia Wang, Yixin Ding, Yunbo Liu, Mingyu Ma, Ji Wang, Zengshun Kou, Shuo Liu, Bo Jiang, Sichuan Hou

**Affiliations:** ^1^ Department of Urology Qingdao Municipal Hospital Qingdao University Qingdao China; ^2^ Department of Oncology The Affiliated Hospital of Qingdao University Qingdao China; ^3^ Department of Urology The Affiliated Hospital of Qingdao University Qingdao China

**Keywords:** biomarkers, CPA4, kidney renal clear cell carcinoma, molecular mechanism, prognosis

## Abstract

Clear cell renal cell carcinoma (ccRCC) is a commonly occurring and highly aggressive urological malignancy characterized by a significant mortality rate. Current therapeutic options for advanced ccRCC are limited, necessitating the discovery of novel biomarkers and therapeutic targets. Carboxypeptidase A4 (CPA4) is a zinc‐containing metallocarboxypeptidase with implications in various cancer types, but its role in ccRCC remains unexplored. The Cancer Genome Atlas (TCGA) and Gene Expression Omnibus (GEO) databases were utilized in order to investigate the differential expression patterns of CPA4. The expression of CPA4 in ccRCC patients was further verified using immunohistochemical (IHC) examination of 24 clinical specimens. A network of protein–protein interactions (PPI) was established, incorporating CPA4 and its genes that were expressed differentially. Functional enrichment analyses were conducted to anticipate the contribution of CPA4 in the development of ccRCC. To validate our earlier study, we conducted real‐time PCR and cell functional tests on ccRCC cell lines. Our findings revealed that CPA4 is overexpressed in ccRCC, and the higher the expression of CPA4, the worse the clinical outcomes such as TNM stage, pathological stage, histological grade, etc. Moreover, patients with high CPA4 expression had worse overall survival, disease‐specific survival and progress‐free interval than patients with low expression. The PPI network analysis highlighted potential interactions contributing to ccRCC progression. Functional enrichment analysis indicated the involvement of CPA4 in the regulation of key pathways associated with ccRCC development. Additionally, immune infiltration analysis suggested a potential link between CPA4 expression and immune response in the tumour microenvironment. Finally, cell functional studies in ccRCC cell lines shed light on the molecular mechanisms underlying the role of CPA4 in promoting ccRCC formation. Overall, our study unveils CPA4 as a promising biomarker with prognostic potential in ccRCC. The identified interactions and pathways provide valuable insights into its implications in ccRCC development and offer a foundation for future research on targeted therapies. Further investigation of CPA4's involvement in immune responses may contribute to the development of immunotherapeutic strategies for ccRCC treatment.

## 1 INTRODUCTION

Renal cell carcinoma (RCC) is a prevalent urological malignancy worldwide, accounting for around 3% of all tumour cases. It affects around 400,000 people each year and has a death rate of roughly 40%, with a significant recurrence rate.^1^ The incidence of RCC has been increasing at a rate of 2% annually over the past 20 years.^2^ In this study, 70% of RCC is ccRCC, which has the greatest mortality.^3^ The main treatment for early stage ccRCC is surgery, which has a survival rate of 60–70%. Despite the use of diverse therapeutic approaches for advanced ccRCC, the 5‐year survival rate remains around 10%, underscoring the unfavourable prognosis associated with this condition.^4^, ^5^ Currently, the molecular mechanisms and development of ccRCC are still unknown, and the lack of reliable biomarkers and molecular targets for clinical practice is realistic.^6^ Finding novel biomarkers and molecular targets is thus crucial for the therapy, early diagnosis and prognostic assessment of ccRCC.

CPA4 is a Zn‐containing metallocarboxypeptidase localized on chromosome 7q32 and has been found to promote the proliferation and migration of tumour cells in a variety of cancers, including breast cancer and colon cancer.^7^, ^8^, ^9^ It was initially identified through mRNA differential display as a gene induced by butyrate in prostate cancer cells (PC‐3).^10^ CPA4 catalyses the release of carboxy‐terminal amino acids, which may be associated with the formation of the tumour microenvironment.^7^ Previous research has shown a link between CPA4 overexpression and the growth, metastasis and invasion of several cancers.^9^, ^11^, ^12^, ^13^, ^14^, ^15^, ^16^ For example, a recent study showed that CPA4 promotes tumour cell proliferation in breast cancer by affecting the ANG1‐CPA4 axis. It may contribute to the prognosis prediction and early diagnosis. However, previous research has not explored the potential clinical value of CPA4 in ccRCC.

This study included an analysis of the expression of CPA4 and its potential association with various clinical characteristics. Furthermore, we verified its expression through IHC of 24 clinical specimens from patients with kidney renal clear cell carcinoma (KIRC). Additionally, we constructed a PPI network involving CPA4 and its associated differentially expressed genes. We predicted the role of CPA4 in promoting the development of KIRC by integrating cell signalling pathway enrichment analysis and immune infiltration analysis. Finally, we aimed to reveal the specific biological mechanism of CPA4 in the development of ccRCC and evaluate its feasibility as a potential therapeutic target.

## 2 TOOLS AND TECHNIQUES

### 2.1 Source of the data and preprocessing

We obtained mRNA expression profile of CPA4 in pan‐cancer and corresponding normal tissues via the TCGA and the GTEx database.^17^ Out of our interest, the RNA‐seq data of unpaired and paired samples in KIRC from the TCGA database were collected and processed subsequently. By searching the published literature and the GEO database, GSE105261, GSE53757 and GSE66271^18^, ^19^ data sets were included in this study. The ‘limma’ and other R (v3.6.3) packages were used for normalization, standardization and visualizations. We further performed multi‐omics analysis to explore the protein expression of CPA4 in ccRCC using the CPTAC on the UALCAN database.^20^

### 2.2 Differential expression analysis of CPA4

The Wilcoxon rank sum test was used to evaluate the differential expression of CPA4 in pan‐cancer. Shapiro–Wilk normality analysis of the expression profile data of CPA4 in paired and unpaired samples was followed by the Wilcoxon rank sum test. The chi‐square test was used to analyse the relationship between CPA4 expression and clinical data of TCGA‐KIRC patients. All the above analyses defined *p* < 0.05 to be statistically significant.

Analysis of differentially expressed genes (DEGs) in CPA4 expression groups with high and low expression CPA4 high or low was characterized statistically as levels of CPA4 expression above or below the median, respectively. The differential expression analysis was conducted with adjusted *p*‐value < 0.05 and |Log2‐fold change| > 1. To visualize the volcano maps for the differential genes that were screened and to analyse the PPI networks, the Cytoscape and the STRING database were used.^21^ The hub gene was screened using MCODE. The hub gene was subsequently employed to illustrate the co‐expression patterns of the CPA4 gene.

### 2.3 Functional enrichment analysis

The most relevant genes about CPA4 were obtained for the GO, KEGG and GSEA functional enrichment analyses. The gene set ‘h.all.v2022.1.Hs.symbols.gmt [Hallmarks]’ was chosen for the GSEA analysis.^22^ We defined the enrichment notability threshold as the false discovery rate (FDR) < 0.25 and *p*.adjust < 0.05.

### 2.4 Immune infiltration analysis of CPA4

The 24 immune cell infiltration level between CPA4‐high and CPA4‐low groups were analysed, and the corresponding enrichment scores were calculated by the ssGSEA algorithm. We defined the threshold of significant relative as the *p*‐value < 0.001.

### 2.5 Clinical statistical analysis, model construction and evaluation of prognosis

Using the Wilcoxon signed rank sum test, we compared CPA4 with clinicopathological characteristics. CPA4 expression levels were analysed to subgroup patients for overall survival (OS), disease‐specific survival (DSS), progress‐free interval (PFI) and other clinical parameters using Cox regression and Kaplan–Meier methods. Multivariate Cox analysis evaluated CPA4 expression and clinical characteristics on survival. Median values established CPA4 expression thresholds.

Using multivariate analysis and the Cox regression model, we created nomogram plots with independent prognostic indicators and predicted survival at 1 year, 3 years and 5 years. Calibration analysis and calibration plots determined nomogram plot prediction accuracy.

### 2.6 Immunohistochemical (IHC)

We collected 24 pairs of ccRCC tumour tissues and paracancerous tissues after ethical approval and informed patient consent. All samples were undergoing the following process: fixing (10% formalin for 24 h), embedding (with paraffin), thin section (2–3 μm) and attaching to glass slides finally. After dewaxing and hydration, immerse in citric acid buffer (PH6.0, MVS‐0100; Fuzhou Maixin Biotechnology Development Co., Ltd.) and cook in a pressure cooker. Afterwards, rinse with PBS washing solution (P1010‐2 L, Soleibao Technology Co., Ltd.). Use the endogenous peroxidase blocker in the mouse/rabbit polymer detection system (PV‐6000, Zhongshan Jinqiao Biotechnology Co., Ltd.) to inactivate endogenous peroxidase, incubate at 37°C for 10 min, and then wash with PBS washing solution. Use animal non‐immune serum (sheep) (SP KIT‐B3; Fuzhou Maixin Biotechnology Development Co., Ltd.) for 20 min at 24°C to prevent non‐specific binding. Rabbit anti‐CPA4 antibody (26824‐1‐AP; 1:300; Proteintech & Wuhan Sanying Bio) was used to stain for 12 h at 4°C. The secondary antibody was an enzyme‐labelled goat anti‐mouse/rabbit IgG polymer (product number PV‐6000, Zhongshan Jinqiao Biotechnology Co., Ltd., stained at 37°C for 20 min). Use the DAB Chromogenic Kit (product code: DAB‐1031, Fuzhou Maixin Biotechnology Development Co., Ltd.) as the chromogen to observe the reaction product and counterstain with 0.1% haematoxylin at room temperature for 2 min. The relevant section photos were captured at either ×200 or ×400 microscopic magnification. The protein expression levels were rated based on the intensity of malignant/epithelial cell staining and the proportion of immunoreactive cells. The IHC score was as follows: unstained tissue = 0, 20% of cells with weak or moderate to strong staining = 1, 20%–40% of cells with moderate or strong staining = 2 and >40% of cells with strong staining = 3.

### 2.7 Cell culture and culture of stably transfected cell lines

The 786‐O cell line (Shanghai iCell Biotechnology Co., Ltd) was cultured in Minimum Essential Medium (RPMI‐1640, iCell‐0002, iCell) supplemented with 10% fetal bovine serum, 1% non‐essential amino acids and 1% penicillin–streptomycin. RPMI‐1640 medium with 1% penicillin–streptomycin and 10% foetal bovine serum grew OS‐RC‐2 cells (Shanghai iCell Biotechnology Co., Ltd). Humidified 37°C and 5% CO2 grew the two cell lines.

We generated the lentiviral vector plasmids pLKO.1‐Scramble and pLKO.1‐shCPA4, respectively, employing the lentiviral vector plasmid pLKO.1‐Puro (FH1717; Hunan Fenghui Biotechnology). shCPA4's interference sequence was 5′‐GTGGTAGATTTCATCCAAA‐3′, whereas shScramble's was 5′‐GTATAAGTCAACTGTTGAC‐3′. This work employed three‐plasmid packaging for lentiviruses. The HEK293T cells (iCell‐h237, iCell) were transfected using a combination of the lentiviral vector plasmid, the packaging plasmids PMD2.G (BR037, Fenway) and psPAX2 (BR036, Fenway), and the transfection reagent Lipofectamine™ 3000 (L3000150, Thermo Fisher). At 48 and 72 h post‐transfection, the supernatant was collected through filtering.

Three hundred thousand cells were seeded per six well plate in this investigation in order to generate stably transfected cell lines. After 24 h, each well added 1 mL of lentivirus media. The medium was changed after 48 h. In 2 mg/mL puromycin, virus‐infected cells were chosen. A week of puromycin selection preceded cell collection and analysis.

### 2.8 Real‐time PCR

The extraction of total RNA was performed on cultured cells using the EasyPure RNA kit (ER10101; TransGen) in accordance with the procedure provided by the manufacturer. An ABI‐Q3 quantitative PCR instrument (Thermo Fisher Scientific, Inc.) was used to conduct PCR. The expression level was determined using the $2^{-\Delta \Delta C_{t}}$ method and adjusted against GAPDH mRNA. The primer sequences of CPA4: 5′‐CCAGATGCCGAGGAAC‐3′ and 5′‐TACGCCCAGTCGATGC‐3′; GAPDH: 5′‐GAAGGTGAAGGTCGGAGTC‐3′ and 5′‐GAAGATGGTGATGATTTC‐3′.

### 2.9 Cell counting kit‐8 assays

The CCK‐8 reagent (10 mL/well; Bioss product no. BA00208) was introduced into each well of a 96‐well plate that had been previously populated with 786‐O cells (3000 cells/well) at time intervals of 24, 48 and 96 h. The measurement of absorbance at 450 nm was conducted for each well using a microplate reader (E0226; Detie, Inc.).

### 2.10 Invasion test

The upper compartment of the Transwell cell chamber was filled with a suspension of 3 × 10^4^ cells (100 mL) in serum‐free RPMI1640 media. The lower compartment was filled with a medium containing 10% FBS (600 mL). The entire setup was incubated at 37°C for a duration of 30 h. The cells that were subjected to invasion were afterwards subjected to staining using a 10% Giemsa solution and were then visualized and captured using a light microscope.

### 2.11 Wound‐healing assay

Cells were inoculated on a six‐well plate at a density of 3 × 10^5^ cells per well. Create linear incisions by gently scraping with a 200 mL pipette tip. The sample should be rinsed with phosphate‐buffered saline (PBS) in order to eliminate any loose cells. Photographs were captured at two time points, specifically 24 and 48 h following the experimental procedure, utilizing a digital camera and a light microscope manufactured by Motic Corporation.

### 2.12 Colony formation test

The two cell lines were distributed evenly over six‐well plates (100 cells/each well). Cell fixation (methanol), staining (10% Giemsa, Biotopoed, China), imaging and counting were performed after the identification of normal colonies. The experiment was conducted on three separate occasions.

## 3 RESULTS

### 3.1 CPA4 expression in KIRC

**FIGURE 1:**
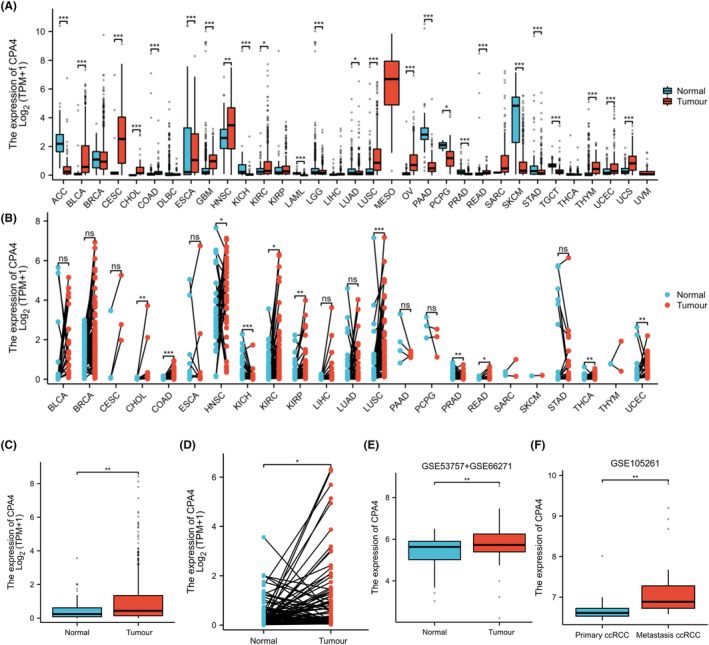
High expression level of CPA4 in pan‐cancer and KIRC tumour tissues. CPA4 expression in (A) unpaired and (B) paired samples from the TCGA database. CPA4 expression in (C) unpaired and (D) paired samples from the TCGA‐KIRC database. Expression of CPA4 in the (E) GSE53757, GSE66271 and (F) GSE105261 databases. ns, *p* ≥ 0.05; *p < 0.05; **p < 0.01; ***p < 0.001.

**FIGURE 2:**
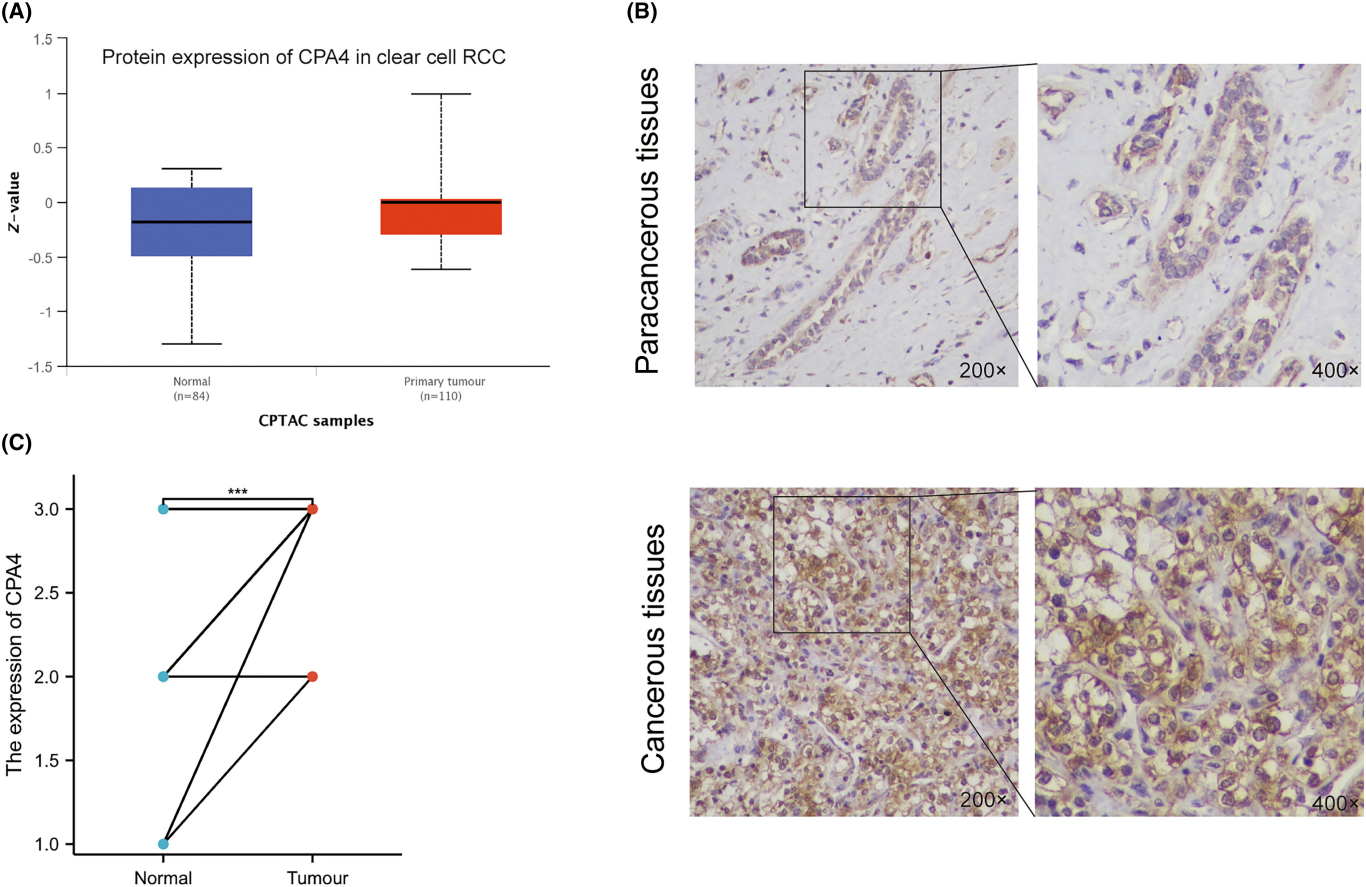
The protein expression of CPA4 in KIRC. (A) The protein expression of CPA4 was higher in KIRC using the CTPAC database; (B) The representative images of IHC staining of CPA4 in KIRC paracancerous tissues and cancerous tissues. (C) The intensity IHC score of 24 KIRC patients' paracancerous and cancerous tissues (Wilcoxon signed rank test, *p* < 0.05).

Firstly, as shown in Figure 1A, we conducted a differential analysis of CPA4 expression in pan‐cancer and discovered that CPA4 expression is high in 14 malignancies, such as Bladder urothelial carcinoma (BLCA) and KIRC (*p* < 0.05). In paired pan‐cancer samples, the expression of CPA4 is increased in various malignancies, including KIRC (Figure 1B). In both paired samples and unpaired samples of the TCGA‐KIRC database (Figure 1C,D), the level of CPA4 expression in tumour tissues was found to be significantly greater compared to normal tissues (*p* < 0.05). Subsequently, we combined the GSE53757 and GSE66271 data sets of the GPL570 platform in the GEO database and found that CPA4 in KIRC was also over‐expressed than that in normal tissues (Figure 1E). In the GSE105261 data set, it was shown that the expression of CPA4 was notably elevated in metastatic ccRCC compared to primary ccRCC, as depicted in Figure 1F. Meanwhile, as shown in Figure 2A, in the CTPAC database, we found that the expression of CPA4 protein was stronger stained in KIRC than in normal tissues. The representative images of IHC staining are shown in Figure 2B. Cancer tissues of ccRCC presented higher IHC staining scores of CPA4 than paracancerous tissues (*p* < 0.05, Figure 2C, Table S1).

### 3.2 Analysis of single‐gene differential expression of CPA4 in KIRC

**FIGURE 3:**
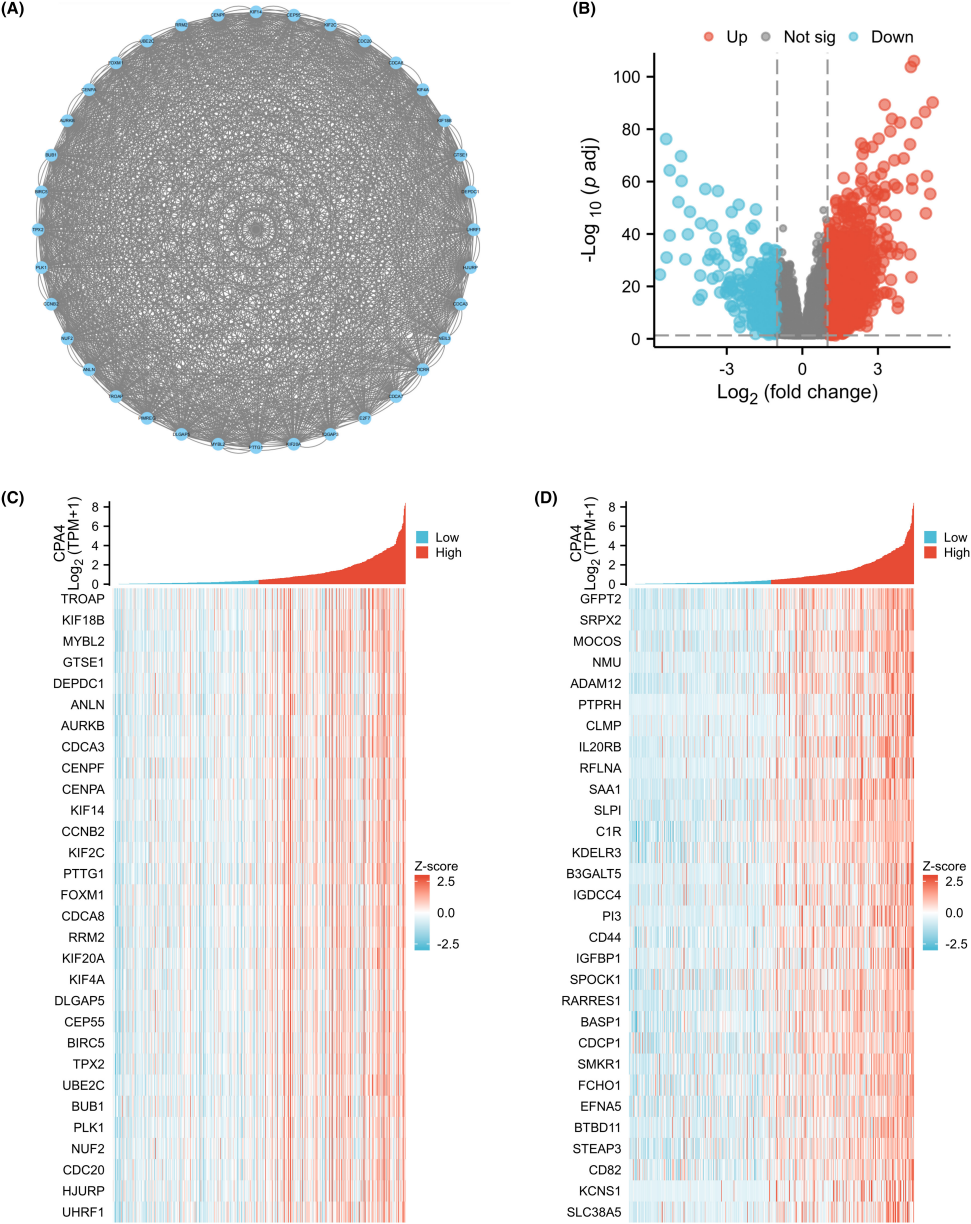
Single gene differential analysis and correlation analysis of CPA4. (A) PPI network of the hub genes; (B) The colour blue is indicative of the expression of genes that have been down‐regulated, whereas the colour red signifies the expression of genes that have been up‐regulated; (C) The heatmap illustrated the co‐expression patterns between hub genes and CPA4; (D) The top 30 genes that had the highest association with CPA4 by a single‐gene correlation study.

After CPA4 single‐gene differential expression analysis, we screened out 1477 genes that met the criteria, and under these criteria, 1119 genes were strongly expressed (positive logFC). There were 358 cases of low expression (negative logFC). The volcano plot was used to depict the single‐gene differential analysis findings (Figure 3B). We constructed distinct protein interaction networks by employing the aforementioned set of 1477 genes. There were 36 HUB genes (TROAP, IF18B, MYBL2, GTSE1, DEPDC1, ANLN, AURKB, CDCA3, CENPF, CENPA, KIF14, CCNB2, KIF2C, PTTG1, FOXM1, CDCA8, RRM2, KIF20A, KIF4A, DLGAP5, CEP55, BIRC5, TPX2, UBE2C, BUB1, PLK1, NUF2, CDC20, HJURP, UHRF1, PIMREG, E2F7, NEIL3, TICRR, QGAP3 and CDCA7) found in all (Figure 3A). The 30 genes with the greatest relationships to CPA4 were then used in a correlation analysis to produce a co‐expression heatmap (Figure 3D). The heatmap illustrating the co‐expression of the HUB genes and CPA4 is depicted in Figure 3C.

### 3.3 Pathway enrichment and analysis of CPA4 in KIRC

**FIGURE 4:**
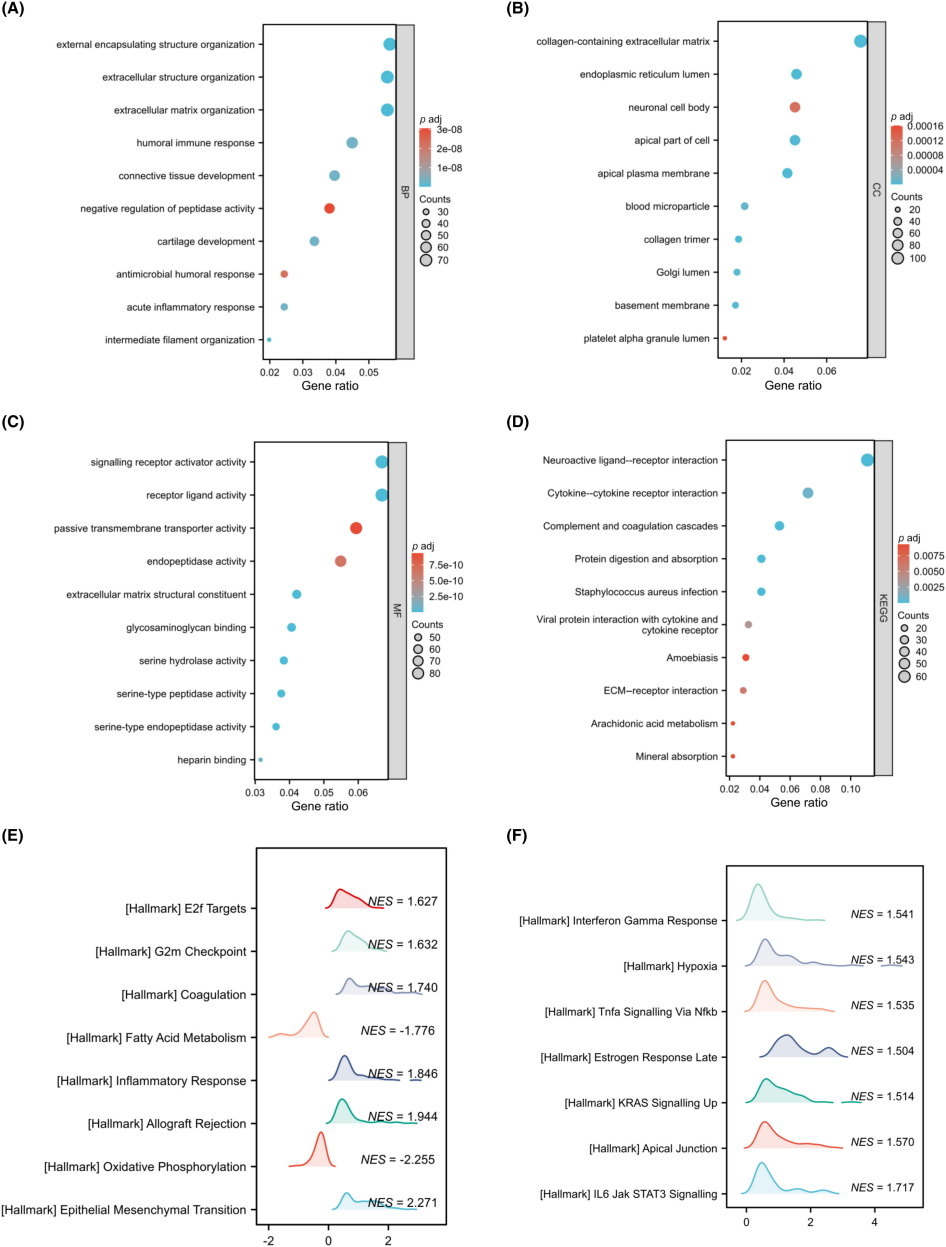
The function enrichment analysis of related DEGs of CPA4 in KIRC. (A–D) GO and KEGG enrichment analysis of related DEGs of CPA4; (E, F) The most significantly enriched pathways between CPA4‐low and CPA4‐high using GESA analysis.

GO enrichment analysis was performed on CPA4 and differential gene (Figure 4A–C). CPA4‐related genes were shown to be involved in external encapsulating structure organization, extracellular matrix organization, endoplasmic reticulum lumen, extracellular matrix structural constituent, serine‐type endopeptidase activity, etc. Subsequently, a KEGG enrichment analysis was performed (Figure 4D). The results showed that CPA4 and staphylococcus aureus infection, protein digestion and absorption and other functions are related.

Finally, we performed GSEA analysis on CPA4 differential genes and selected pathways, and the findings revealed that 15 pathways in all were considerably enriched, namely epithelial–mesenchymal transition, oxidative phosphorylation, allograft rejection, inflammatory response, fatty acid metabolism, etc. (Figure 4E,F).

### 3.4 Immune infiltration and CPA4 expression in a relationship

**FIGURE 5:**
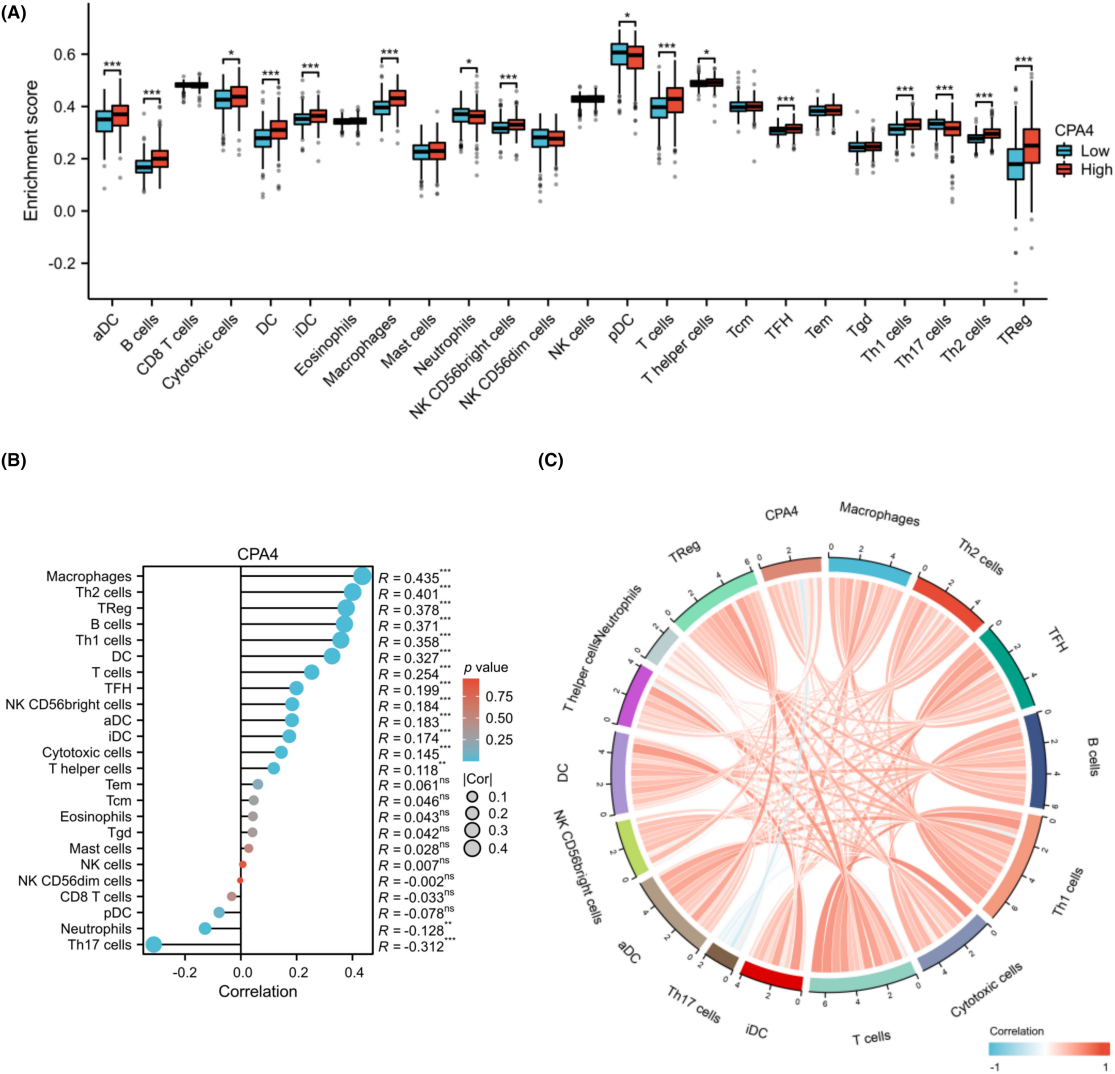
Immune infiltration analysis of CPA4. (A) The enrichment scores of 24 immune cells were compared between CPA4‐high and CPA4‐low groups, and the results were shown using box plots; (B) The lollipop plot represented the correlation between 24 immune cells and CPA4; (C) The chord diagram served to illustrate the correlation, where blue lines indicated negative correlation and red lines indicated positive correlation. ns, *p* ≥ 0.05; *p < 0.05; **p < 0.01; ***p < 0.001.

CPA4 expression is positively related to Macrophages, Th2 cells, Treg, B cells, Th1 cells and other immune cells but negatively related to Th17 cells and neutrophils (Figure 5B, *p* < 0.05). Furthermore, statistically significant difference existed in the immune infiltration scores between the groups with high and low expression levels of CPA4 in macrophages, Th2, Treg, B cells, Th1, DC, T cells, iDC, TFH, aDC, NK CD56 bright cells, Cytotoxic cells, Th17, T helper cells and neutrophils (*p* < 0.05, Figure 5A). Next, we plotted the scatter plot of each immune cell infiltration score with CPA4 expression. The findings exhibiting a significant connection (*p* < 0.001) are given in Figure S1. We produced the correlation chord diagram based on the CPA4 expression and each immune cell infiltration score (Figure 5C). The aforementioned findings showed a strong correlation between the tumour's immune activation state and the high expression of CPA4.

### 3.5 Clinicopathological factors and CPA4 expression correlation

**FIGURE 6:**
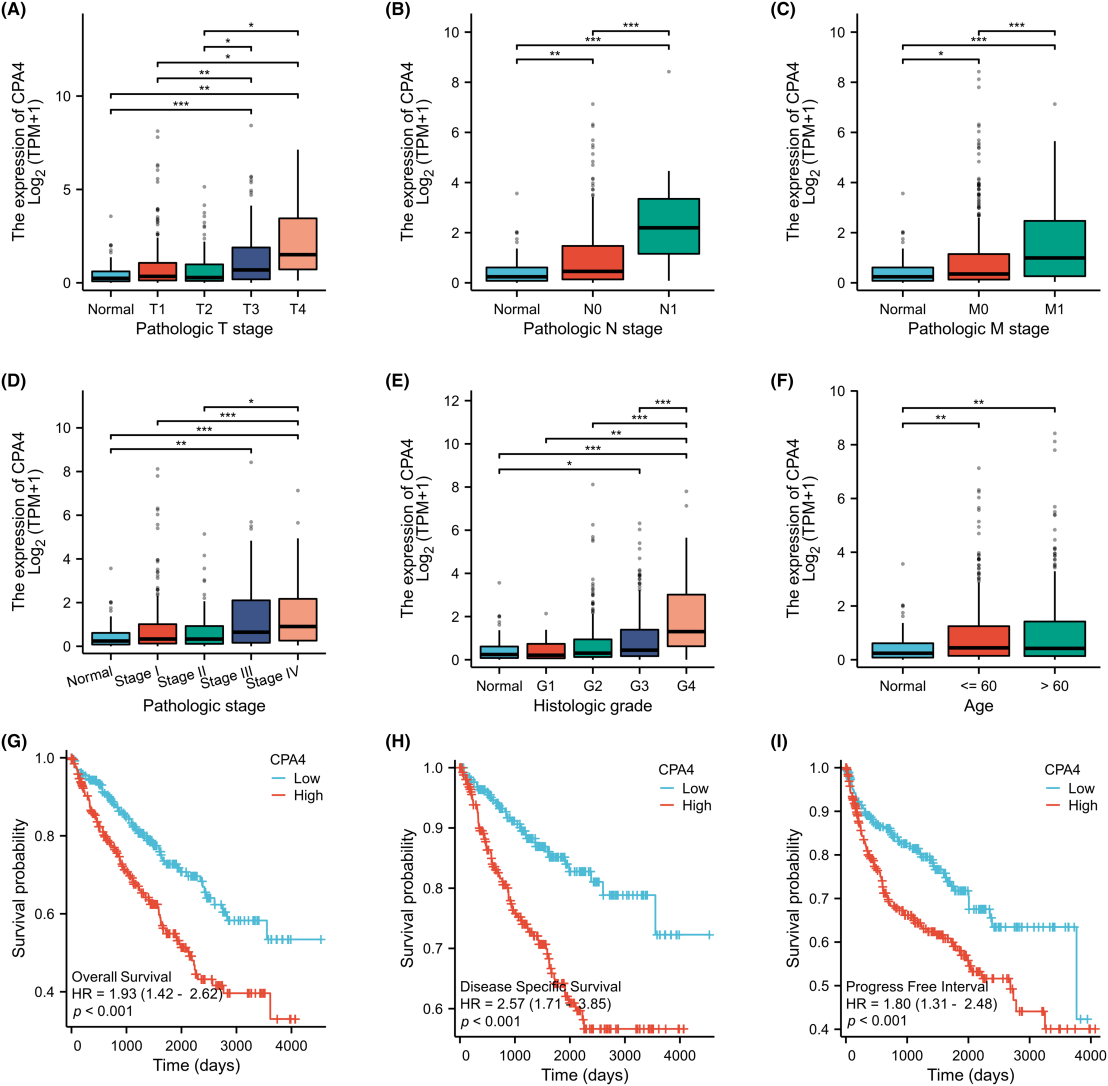
In the TCGA‐KIRC database, the relationship between the expression of CPA4 and clinicopathological cactors. (A–F) The relationship between the expression of CPA4 and six clinicopathological variables. (G–I) K‐M analysis of OS, DSS and PFI between CPA4‐low and CPA4‐high in KIRC. ns, *p* ≥ 0.05; *p < 0.05; **p < 0.01; ***p < 0.001.

The clinical baseline information table (Table 1) was obtained from 613 KIRC samples from the TCGA database to examine CPA4 expression and clinical‐pathological features. Figure 6A–F shows In TNM staging, CPA4 expression and clinical data correlation did not alter substantially. T3 and T4 stages still expressed more than T1 and T2 stages (*p* < 0.05). N1 and M1 patients had greater CPA4 expression than N0 and M0 in both N and M stages *(p* < 0.001).

**TABLE 1:** The clinical baseline information table about the association between the expression of CPA4 and different clinical–pathological characteristics of KIRC patients from the TCGA database.

Characteristics	Low expression of CPA4	High expression of CPA4	*p* Value
*n*	270	271	
Pathologic T stage, *n* (%)			
T1	154 (28.5%)	125 (23.1%)	0.001
T2	41 (7.6%)	30 (5.5%)	
T3	73 (13.5%)	107 (19.8%)	
T4	2 (0.4%)	9 (1.7%)	
Pathologic *N* stage, *n* (%)			
N0	120 (46.5%)	122 (47.3%)	< 0.001
N1	1 (0.4%)	15 (5.8%)	
Pathologic M stage, *n* (%)			
M0	227 (44.7%)	202 (39.8%)	0.001
M1	26 (5.1%)	53 (10.4%)	
Pathologic stage, *n* (%)			
Stage I	154 (28.6%)	119 (22.1%)	0.002
Stage II	33 (6.1%)	26 (4.8%)	
Stage III	54 (10%)	69 (12.8%)	
Stage IV	29 (5.4%)	54 (10%)	
Primary therapy outcome, *n* (%)			
PD	7 (4.8%)	4 (2.7%)	0.109
SD	6 (4.1%)	0 (0%)	
PR	2 (1.4%)	0 (0%)	
CR	72 (49%)	56 (38.1%)	
Gender, *n* (%)			
Female	96 (17.7%)	91 (16.8%)	0.629
Male	174 (32.2%)	180 (33.3%)	
Race, *n* (%)			
Black or African American	32 (6%)	25 (4.7%)	0.493
White	232 (43.4%)	237 (44.4%)	
Asian	3 (0.6%)	5 (0.9%)	
Age, *n* (%)			
≤60	132 (24.4%)	137 (25.3%)	0.699
>60	138 (25.5%)	134 (24.8%)	
Histologic grade, *n* (%)			
G1	10 (1.9%)	4 (0.8%)	<0.001
G2	135 (25.3%)	101 (18.9%)	
G3	102 (19.1%)	105 (19.7%)	
G4	16 (3%)	60 (11.3%)	
Serum calcium, *n* (%)			
Low	105 (28.6%)	99 (27%)	0.309
Normal	71 (19.3%)	82 (22.3%)	
Elevated	3 (0.8%)	7 (1.9%)	
Haemoglobin, *n* (%)			
Low	124 (26.9%)	140 (30.4%)	0.762
Normal	96 (20.8%)	96 (20.8%)	
Elevated	2 (0.4%)	3 (0.7%)	
Laterality, *n* (%)			
Left	127 (23.5%)	126 (23.3%)	0.931
Right	143 (26.5%)	144 (26.7%)	

### 3.6 The suggestive significance of CPA4 for the prognosis of KIRC patients

**FIGURE 7:**
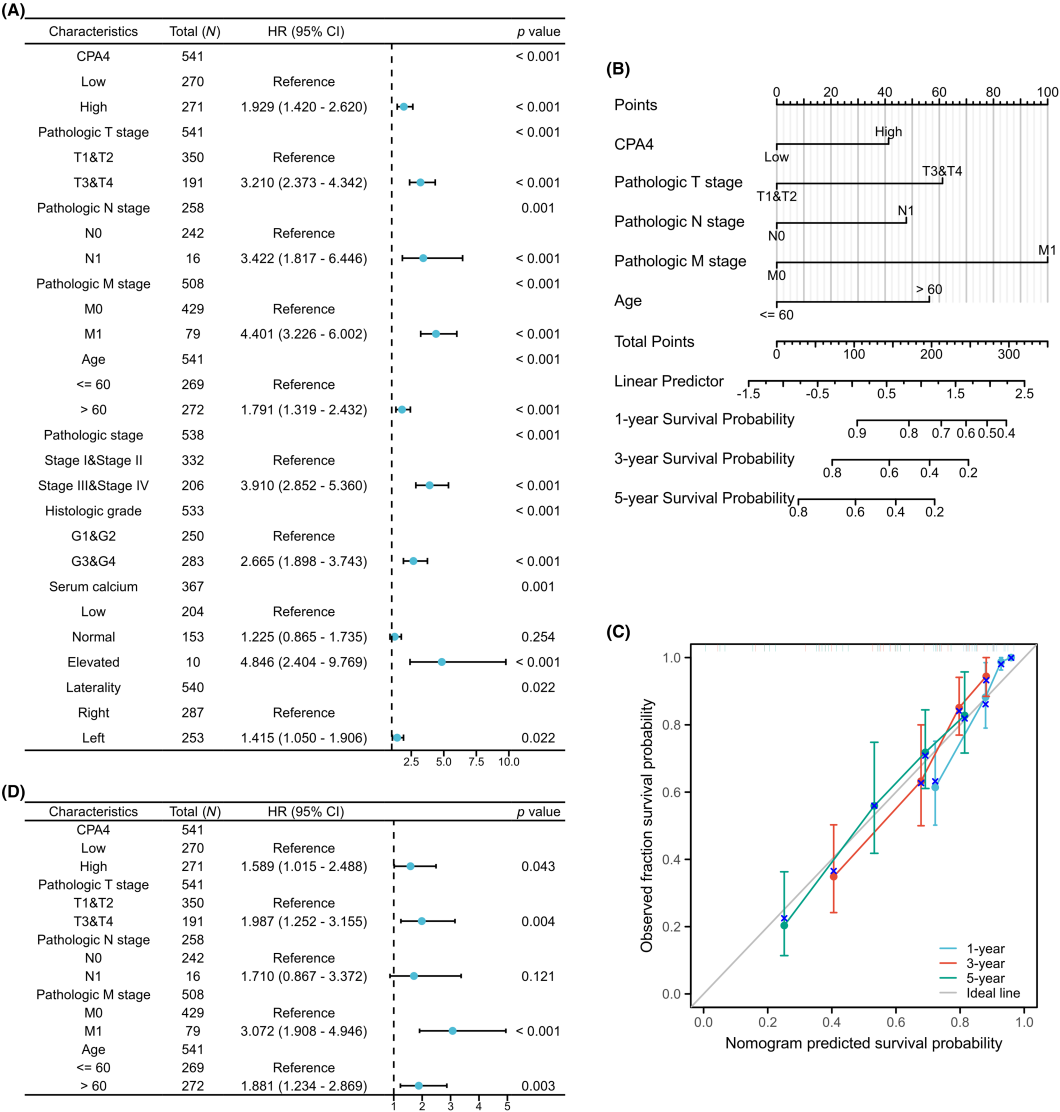
Construction of prognostic nomogram model. Forest map based on (A) univariate and (D) multivariate Cox analyses for OS; (B) A nomogram for predicting 1‐, 3‐ and 5‐year OS survival of KIRC. (C) The calibration curve for the 1‐, 3‐ and 5‐year OS survival nomogram.

CPA4‐high group exhibited a comparatively inferior OS rate (HR = 1.93, *p* < 0.001, Figure 6G). The survival findings of DSS (HR = 2.57, *p* < 0.001) and PFI (HR = 1.80, *p* < 0.001) (Figure 6H,I) show that tumour patients with high CPA4 expression were at risk. Figure S2 shows the findings of survival analysis for various subgroups. CPA4 high expression subgroups are connected with worse survival rates. Finally, we performed Univariate and multivariate Cox regression for common clinical‐pathological factors (Figures 7A,D). TNM stage, age, pathologic stage, histologic grade, laterality, serum calcium and CPA4 were statistically different in the univariate analysis. The statistically significant results were chosen for further investigation, leading to the execution of a multivariate Cox regression analysis. The findings of this analysis revealed that T stage, M stage, age and CPA4 remained statistically significant (*p* < 0.05), suggesting that elevated CPA4 expression independently contributes to the risk of overall survival in patients with KIRC.

### 3.7 CPA4 nomogram construction and verification

Based on the multivariate Cox regression analysis, we created a prognostic nomogram using TNM stage, age and CPA4 to quantify the prognosis of KIRC patients with 0.773 (0.750–0.796) C‐index, indicating moderate accuracy (Figure 7B). After that, we produced the calibration graph in Figure 7C to test the model's prediction accuracy. The deviation correction line is near the ideal curve (45°), and the projected value matches the actual value.

### 3.8 Upregulation of CPA4 enhanced the proliferation, migration and invasion capabilities of KIRC cells

**FIGURE 8:**
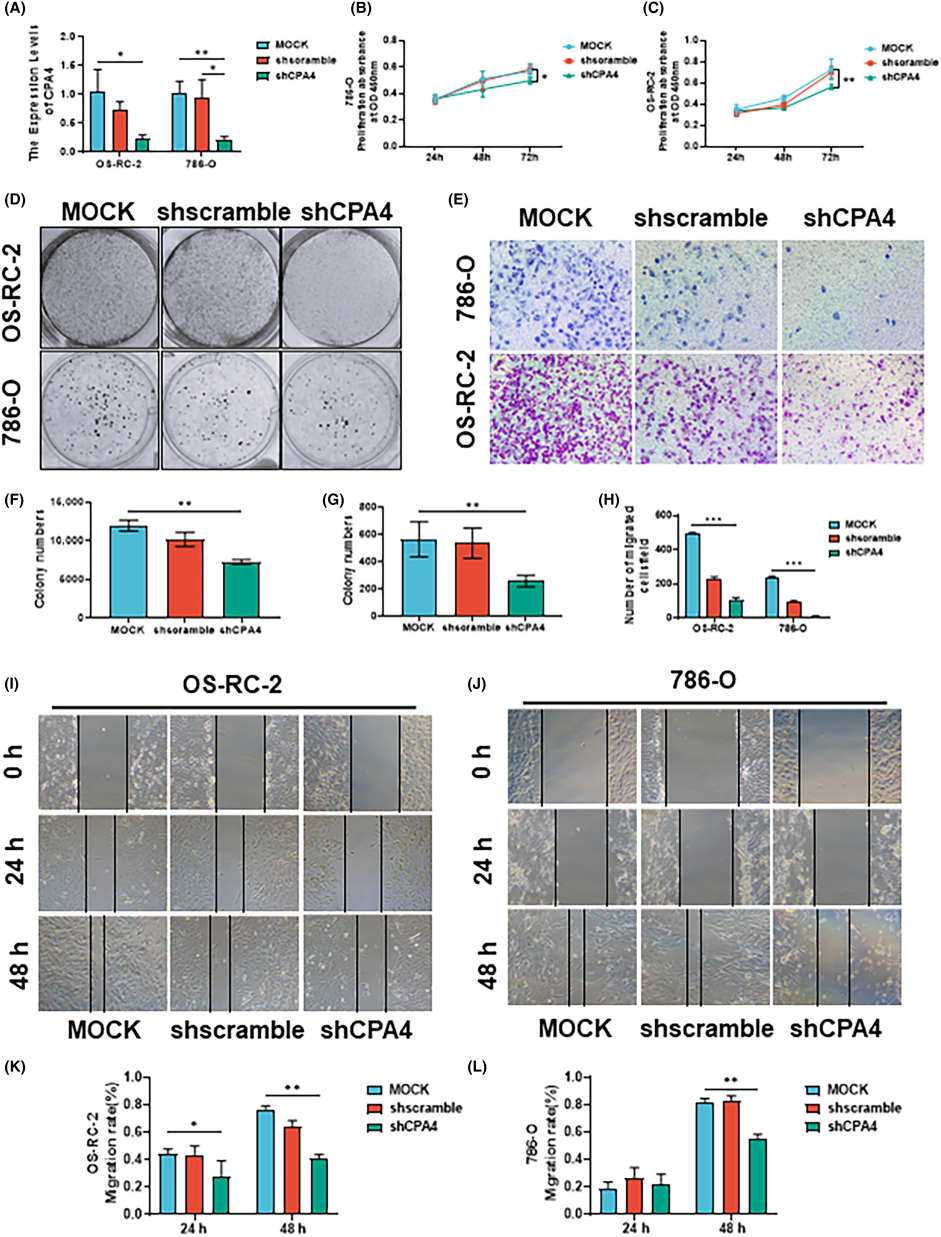
Upregulation of CPA4 enhanced the proliferation, migration and invasion capabilities of KIRC cells. (A) The expression of CPA4 was seen to be considerably reduced following the transfection of shCPA4 in two distinct KIRC cell lines by qRT‐PCR. Downregulation of CPA4 inhibited the proliferation (B–D, F, G), migration (E, H) and invasion (I–L) capabilities of KIRC cells. ns, *p* ≥ 0.05; *p < 0.05; **p < 0.01; ***p < 0.001.

CPA4 expression was verified to be drastically decreased after being knocked down in two cell lines (Figure 8A). Then, using CCK‐8 tests, we discovered that cell proliferation was considerably decreased when CPA4 was knocked down (Figures 8B,C). In addition, Transwell experiments were conducted to assess the impact of CPA4 knockdown on cellular invasion capacity, revealing a noteworthy and statistically significant reduction (Figure 8E–H). Furthermore, we conducted investigations on wound healing and observed a significant decrease in the metastatic potential of cells with suppressed CPA4 expression compared to the control group, as the duration of the study progressed (Figure 8I–L). Finally, the colony formation assay displayed that after knocking down the expression of CPA4 in cells, their colony formation ability decreased (Figures 8D,F,G).

## 4 DISCUSSION

Over 90% of kidney malignancies are RCC, which originates from the renal epithelium.^23^ KIRC, a subtype of kidney cancer associated with high death rates, invasion and metastasis, has been found to be insensitive to chemotherapy or radiotherapy.^24^, ^25^ Hence, it is crucial to explore alternative molecular indicators to identify and predict the presence and prognosis of KIRC, facilitating the development of a more tailored and effective treatment strategy.

We observed that CPA4 was highly expressed in KIRC and dramatically increased in metastatic KIRC patients, which indicates that CPA4 may play a key role in the occurrence and development of KIRC. In the CPTAC database, the expression of CPA4 protein was also higher in KIRC than in normal kidney tissues. We verified this by IHC in cancerous and paracancerous tissues of 24 KIRC patients. The expression of CPA4 in the M1 and N1 stages was considerably greater than that in the M0 and N0 stages (*p* < 0.05), suggesting that high CPA4 expression was strongly connected to the incidence, development, metastasis and invasion in KIRC. This is consistent with the results of our in vitro cell experiments. In research similar to this one, Wei et al. found CPA4 as a molecular diagnostic marker for immune infiltration in bladder cancer.^15^

Furthermore, our study elucidated the molecular mechanisms through which CPA4 promotes the progression of KIRC by the PPI network. This investigation identified 36 hub genes associated with CPA4 expression, including Kinesin superfamily proteins (KIFs), CENPs, TROAP, GFPT2, BUB1 and others. KIFs are intracellular transport system hub proteins that are critical for cell function and shape.^26^ Numerous cellular processes, including mitosis, meiosis and the transport of macromolecules (such as axonal transport), are supported by the active movement of kinesins.^27^ A change in kinesin expression or function may result in carcinogenesis since mitosis is complex and tightly controlled.^28^ Numerous studies have correlated KIF18B with the proliferation, migration and invasion of cancer cells,^29^, ^30^, ^31^, ^32^ whereas KIF14 has been implicated in the development of gastric, colorectal and lung cancers.^33^, ^34^, ^35^ Therefore, we propose that CPA4 may accelerate the development of ccRCC by promoting the activity of specific KIF‐associated proteins. Centromere proteins (CENPs) are essential for chromosome segregation during mitosis and meiosis in eukaryotic cells.^36^ BUB1 is a multidomain paralog that plays crucial functions in the spindle assembly checkpoint (SAC) and chromosomal alignment during mitosis.^37^ In addition, GFPT2 and SRPX2, which are closely related to CPA4, also promote the progression of various cancers.^38^, ^39^ These results suggest a function for CPA4 in cell division and cell cycle control, hence playing a key role in the progression and dissemination of KIRC.

To better understand the molecular processes associated with CPA4 in tumour growth, functional enrichment analyses were performed. The findings of the study indicate that CPA4 contributes to the organization of the extracellular matrix, humoral immune response, connective tissue development, endoplasmic reticulum lumen, extracellular matrix structural constituent and other biological processes. Studies suggest that the extracellular matrix (ECM) may be critical to metastasis. During tumour progression, The composition and content of the ECM are influenced by biophysical and biological factors and have significant effects on the characteristics of tumour and stromal cells, which include the regulation of cell proliferation and motility.^40^ Cancer cells proliferate, survive and exhibit enhanced invasive properties as a result of extensive biochemical signalling and biomechanical changes in the ECM, which are important in tumour biology.^41^ The humoral immune response is increasingly related to cancer. Several studies found autoantibodies, including anti‐p53, anti‐MUC1 and anti‐CA125, to be promising cancer biomarkers, particularly in panels.^42^ One of the proteins belonging to the CCN family, which is released and associated with the extracellular matrix, plays a role in the processes of angiogenesis and tumour development. Therefore, the formation of connective tissue may control the invasion, angiogenesis and anoikis of cancer cells.^43^ The moving endoplasmic reticulum (ER) performs numerous vital cellular processes. Because cancer cells need to reuse their organelles to proliferate, ER stress may promote autophagy in cells.^44^ The KEGG results also revealed that CPA4 was considerably enriched in ECM–receptor interaction. This observation aligns with the results obtained from the GO investigation.

GSEA results indicated that CPA4 was closely related to epithelial–mesenchymal transition (EMT), oxidative phosphorylation (OXPHOS) and other epigenetic genes which demonstrate a close association with tumour cells. EMT contributes to tumorigenesis in the aspect of mobility, invasion abilities and anti‐apoptosis, providing favourable conditions for tumour cells.^45^ EMT gives cancer cells in the setting of neoplasms higher tumour‐initiating and metastatic potential as well as increased resistance to being removed by various therapy regimens.^46^ Thus, increased CPA4 expression may accelerate ccRCC metastasis, invasion and migration, supporting the prior findings.

Furthermore, there was a positive correlation observed between the expression of CPA4 and the invasion of macrophages, Th2 cells, Treg cells, B cells, Th1 cells and DCs, while negatively related to the infiltration of Th17 cells and neutrophils. The presence of macrophages in solid tumours is often associated with treatment resistance and poor prognosis.^47^ The shift from Th1 to Th2 dominance accelerates the immunosuppressive response in the tumour microenvironment, which was consistent with the positive correlation between CPA4 expression and Th2 cell infiltration.^48^, ^49^ Th17 cells, on the other hand, play a powerful role in antitumor immunity, suggesting that increasing the Th17/Treg ratio may benefit patients with aggressive tumours.^50^ Since CPA4 presented the opposite relationship to Treg and Th17 cells, this could have negative implications for the treatment of aggressive tumours. The aforementioned findings suggest that elevated levels of CPA4 are unfavourable for the immune response against tumours and can hinder the effectiveness of tumour immunotherapy. This may contribute to an increased likelihood of developing resistance to cancer treatment drugs.

We investigated the prognostic value of CPA4 by examining its correlation with clinicopathological characteristics using the TCGA database. Strong associations were seen between the expression of CPA4 and the TNM stage, pathological stage and histological grade of the tumour. High CPA4 expression was associated with a higher likelihood of lymphatic and distant metastases, indicating an unfavourable prognosis for KIRC patients. Survival analyses revealed that patients with high CPA4 expression had significantly shorter OS, PFI, and DSS. The results were consistent in the subgroup prognostic analyses. The nomogram map was created as a clinical prognostic prediction tool using the multivariate Cox regression findings at the same time, and the model's accuracy was examined. The calibration figure shows that there was excellent agreement between the actual OS values at 1 year, 3 years and 5 years and the anticipated values. As a result, the nomogram created for this research may end up becoming a brand‐new and useful prognostic prediction tool. This suggests that it could serve as a potential biomarker that could provide important information for early diagnosis and treatment selection.

Finally, this study used two KIRC cell lines to down‐regulate the expression of CPA4 by stably transfecting shRNA. The expression of CPA4 was down‐regulated, thereby reducing the viability of KIRC cells and the ability of cell proliferation, metastasis and invasion. This finding further confirms that CPA4 is involved in the KIRC cell cycle, promotes the occurrence and growth of KIRC and contributes to the invasion and metastasis of KIRC cells. It provides a potentially reliable site for tumour‐targeted therapy.

In conclusion, CPA4 overexpression could inhibit the antitumor immune response while promoting the incidence, metastasis, and invasion of KIRC. The presence of CPA4 demonstrates a correlation and potential for predicting the occurrence of KIRC. All of which will aid medical professionals in developing better patient‐friendly treatment regimens.

There are certain restrictions on this research. Although this study provides important preliminary findings, its sample size is relatively small, and future validation of these results in a broader patient population is needed. It is uncertain whether the use of a single biomarker would provide enough accuracy for prediction and diagnosis. Hence, future research will need to focus on combinations of multiple distinct biomarkers. In addition, the molecular mechanism of how CPA4 regulates ccRCC cell behaviour remains to be further explored.

## AUTHOR CONTRIBUTIONS

**Kongjia Wang:** Data curation (lead); methodology (lead); resources (lead); software (lead); writing – original draft (lead). **Yixin Ding:** Formal analysis (lead); writing – original draft (equal). **Yunbo Liu:** Investigation (equal); methodology (supporting); software (supporting); writing – review and editing (supporting). **Mingyu Ma:** Formal analysis (supporting); resources (supporting); supervision (supporting); validation (supporting). **Ji Wang:** Funding acquisition (supporting); validation (supporting); writing – review and editing (supporting). **Zengshun Kou:** Data curation (supporting); methodology (supporting). **Shuo Liu:** Resources (supporting); software (supporting). **Bo Jiang:** Funding acquisition (equal); supervision (lead); writing – review and editing (supporting). **Sichuan Hou:** Funding acquisition (lead); supervision (equal); writing – review and editing (equal).

## CONFLICT OF INTEREST STATEMENT

The final version submitted has been reviewed and approved by all authors of this research. The authors have disclosed that they have no potential conflicts of interest pertaining to the research and publishing of this article.

## Supporting information


Table S1

Figure S1

Figure S2

## Data Availability

The datasets generated and analysed during the current study are available in the GEO (https://www.ncbi.nlm.nih.gov/geo/), TCGA (https://portal.gdc.cancer.gov), and NALCAN (https://ualcan.path.uab.edu/) repositories.
