# Supplementary material for: CPA4 as a biomarker promotes the proliferation, migration and metastasis of clear cell renal cell carcinoma cells
Source: J Cell Mol Med. 2024 Mar 17;28(7):e18165. doi: 10.1111/jcmm.18165 (PMC10945090; doi:10.1111/jcmm.18165)
Supplement: Supplementary file 1 — Table S1 [file JCMM-28-e18165-s001.docx]

**Supplemental table 1. IHC scores of Tumor tissues and paracancerous tissues in 24 KIRC patients**

| Patient ID | IHC scores | |
| --- | --- | --- |
|  | Cancerous tissues | Paracancerous tissues |
| 1 | 3 | 3 |
| 2 | 3 | 2 |
| 3 | 3 | 2 |
| 4 | 3 | 3 |
| 5 | 2 | 2 |
| 6 | 3 | 2 |
| 7 | 3 | 3 |
| 8 | 3 | 3 |
| 9 | 3 | 2 |
| 10 | 3 | 3 |
| 11 | 3 | 2 |
| 12 | 3 | 2 |
| 13 | 3 | 1 |
| 14 | 2 | 1 |
| 15 | 3 | 1 |
| 16 | 3 | 1 |
| 17 | 3 | 2 |
| 18 | 3 | 2 |
| 19 | 3 | 3 |
| 20 | 3 | 3 |
| 21 | 3 | 3 |
| 22 | 3 | 3 |
| 23 | 3 | 3 |
| 24 | 3 | 2 |
