# Supplementary figures and images for: CPA4 as a biomarker promotes the proliferation, migration and metastasis of clear cell renal cell carcinoma cells
Source: J Cell Mol Med. 2024 Mar 17;28(7):e18165. doi: 10.1111/jcmm.18165 (PMC10945090; doi:10.1111/jcmm.18165)

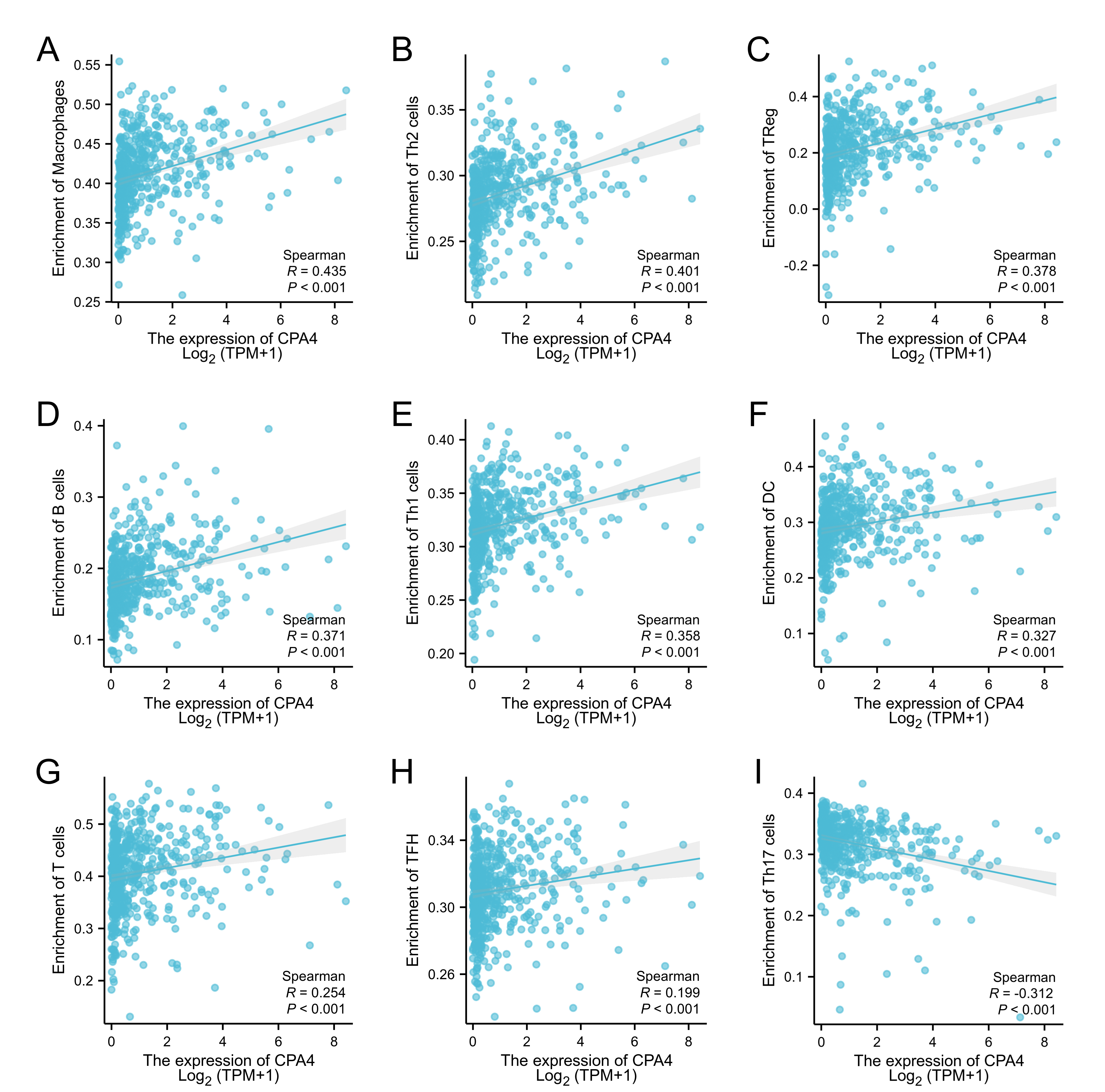

Supplement: Supplementary file 2 — Figure S1 [file JCMM-28-e18165-s002.tiff]

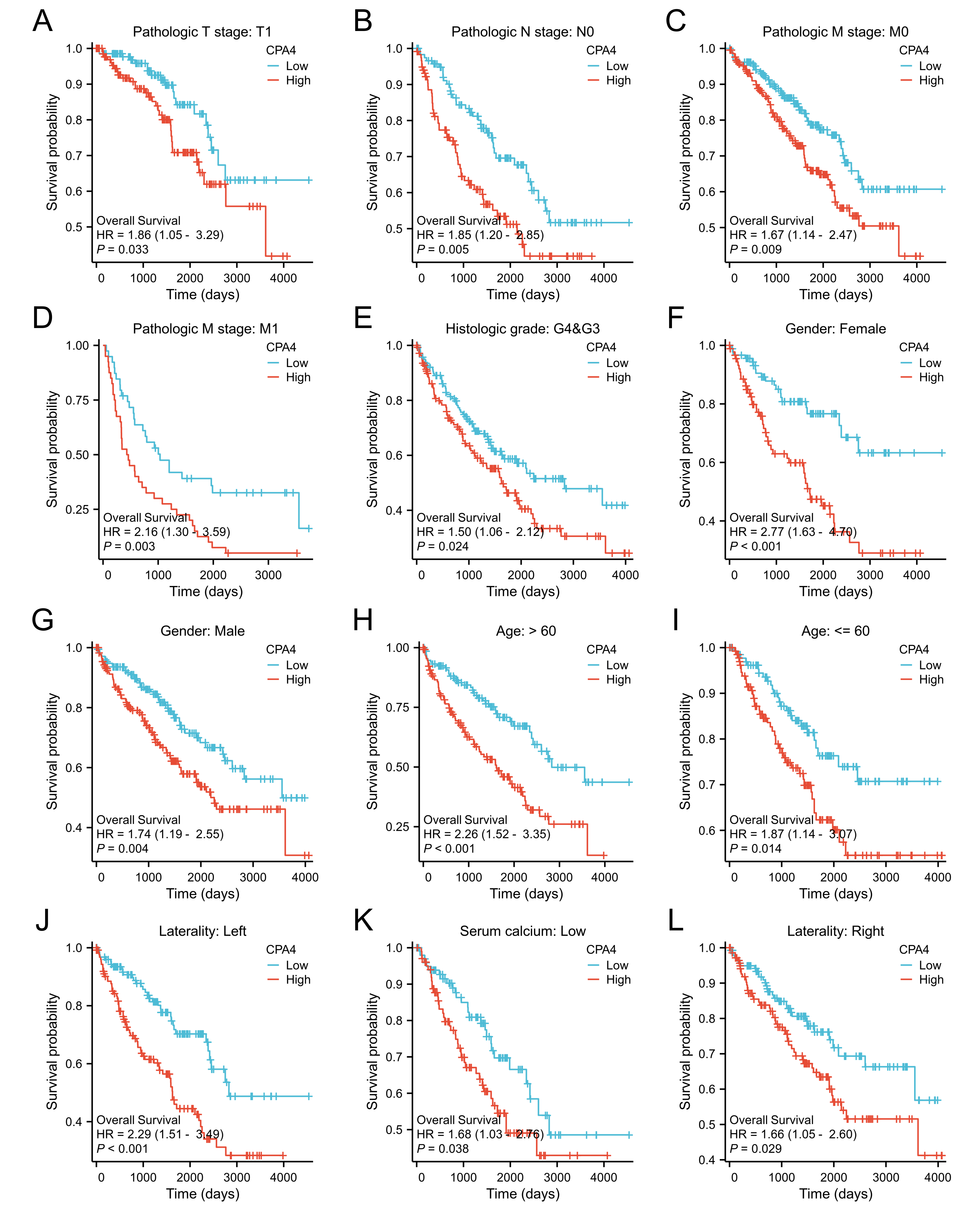

Supplement: Supplementary file 3 — Figure S2 [file JCMM-28-e18165-s003.tiff]
